# Assessment of Components Associated with Average Daily Gain of Finishing Lambs Fed with Two Roughage Sources Using Integrative Metabolomics

**DOI:** 10.3390/ani16091360

**Published:** 2026-04-29

**Authors:** Junnan Ma, Shuzhen Wang, Daiyi Yang, Xiaodong Chen, Yan Tu, Tao Ma

**Affiliations:** 1 Key Laboratory of Feed Biotechnology of the Ministry of Agriculture and Rural Affairs, Institute of Feed Research of Chinese Academy of Agricultural Sciences, Beijing 100081, China; majunnan@caas.cn (J.M.); wsz20030105@163.com (S.W.); sxauydy@163.com (D.Y.); tuyan@caas.cn (Y.T.); 2 College of Animal Science and Technology, Ningxia University, Yinchuan 750021, China; isxiaodong@163.com

**Keywords:** average daily gain, extruded rapeseed straw, Hu lamb, integrative metabolomics

## Abstract

Rapeseed straw and peanut vine are abundant agricultural by-products in China, but their low digestibility has largely restricted their application in ruminant production. Extrusion processing has been shown to improve the fiber structure and nutrient availability of roughages, yet the underlying metabolic mechanisms by which extruded rapeseed straw (ERS) influences growth performance remain insufficiently elucidated. This study aimed to explore the metabolic mechanisms of average daily gain (ADG) affected by rapeseed straw feeding through studying metabolites from four biological matrices (rumen fluid, serum, liver and muscle) collected from 24 Hu lambs fed extruded rapeseed straw (ERS, *n* = 12) and peanut vine hay (PVH, *n* = 12). The Hu lambs fed ERS exhibited greater ADG during the late finishing stage (60–90 d) than those fed PVH (*p* = 0.03). A total of 666, 274, 147, and 96 metabolites were identified in the rumen fluid, liver, serum and muscle, respectively. In addition, nine, 12, seven, and three significantly different metabolites (VIP > 1 and *p* < 0.05) related to eight significant pathways (starch and sucrose metabolism, galactose metabolism, glyoxylate and dicarboxylate metabolism, glycerophospholipid metabolism, phenylalanine, tyrosine and tryptophan biosynthesis, phenylalanine metabolism, Gly, Ser, and Thr metabolism, arginine biosynthesis, and purine metabolism) were identified in the four biological matrices between the ERS- and PVH-fed Hu lambs. Further integrated key metabolic pathway analysis showed that the ERS-fed Hu lambs may have more comprehensive amino acid and energy metabolisms with relatively fewer carbohydrate metabolisms, suggesting enhancing protein deposition and energy utilization efficiency with associated metabolites and pathways serve as key biomarkers for a higher ADG of Hu lambs when fed ERS.

## Simple Summary

This study explored the regulatory mechanism underlying the improved average daily gain of finishing Hu lambs fed with either extruded rapeseed straw or peanut vine as roughage source. Using a metabolomics approach across multiple biological matrices, we found that feeding extruded rapeseed straw improved average daily gain by optimizing rumen fermentation, enhancing amino acid metabolism, and increasing energy utilization efficiency. These metabolic changes promoted protein deposition and coordinated physiological responses across the rumen, serum, liver, and muscle. The results demonstrate that extruded rapeseed straw can be used as a high-quality roughage to improve growth, supporting the efficient utilization of agricultural straw resources in ruminant production.

## 1. Introduction

China has faced an annual forage shortage of nearly 50 million tons in recent years, with this deficit expected to reach approximately 80 million tons by 2035. Given this situation, exploring alternatives to traditional roughage may be the key to reducing production costs and diversifying the diets of livestock, particularly those of ruminants [1].

Rapeseed straw (*Brassica napus* subsp. *napus*), the main by-product of the rapeseed industry, is rich in nutritional components such as crude fiber, crude protein and crude fat, making it a high-quality forage resource with great potential [2]. China is the leading producer of rapeseed with a large abundance of rapeseed straw (~21 million tons per year), while its comprehensive utilization rate remains relatively low (~25%) [3]. Straw-based roughage has abundant lignocellulose; this tough complex carbohydrate is difficult to digest for humans or monogastric animals. Ruminants have a specialized gut microbiota that can efficiently break down lignocellulose [4], which also makes rapeseed straw a promising feed resource for ruminants. However, the presence of anti-nutritional factors such as glucosinolates and sinapine may limit the voluntary intake of rapeseed straw by ruminants. To overcome these challenges, extrusion is a widely adopted pretreatment technology that breaks down the lignocellulosic structure into defibration and shortening of the fibers in the straws for extruded straw forage [5,6]. Compared with untreated straw, extruded straw forage has significantly increased nutrient contents and exerts positive effects on the growth performance and rumen fermentation of ruminants [7]. Peanut (*Arachis hypogaea* L.) vine is another by-product roughage source that is widely used in ruminants as a replacement for alfalfa and barley to reduce the costs in intensive livestock farming [8]. However, knowledge of the physiological and metabolic mechanisms involved remains limited.

Hu lambs, a native Chinese breed originating from the Taihu Lake region, is characterized by a high reproductive rate, rapid growth performance, and strong environmental adaptability [9,10]. With the development of the economy and improvement in living standards, Chinese demand for lamb meat is increasing year after year. In the face of this challenge, improving the production of lamb is essential for maintaining a stable and sustainable supply of lamb meat [11]. Average daily gain (ADG), which is closely associated with body weight gain, plays a significant role in production practice, like the lamb meat supply of Hu lambs. In our previous study, extruded rapeseed straw feeding was confirmed to improve the ADG of late finishing (60–90 days) Hu lambs compared with peanut vine hay feeding [2]. The underlying mechanisms, particularly the regulatory effects on the ADG of key components, have remained unexplored.

Therefore, in this study, we aimed to elucidate the metabolic mechanisms regarding how the extruded rapeseed straw feeding impacts ADG by studying the metabolite profiles of four biological matrices in Hu lambs. GC-TOF/MS-based metabolomics was performed to investigate the simultaneous responses of the rumen fluid, serum, liver and muscle to two different diets to establish the correlations among the biological matrices’ metabolisms and to gain insights into the mechanisms underlying extruded rapeseed straw feeding related to ADG in late finishing Hu lambs. We hypothesized that extruded rapeseed straw improves growth performance in Hu lambs by modulating the multi-tissue metabolic profiles related to energy utilization and nutrient metabolism. This study was conducted to verify this hypothesis and clarify the associated metabolic mechanisms.

## 2. Materials and Methods

### 2.1. Animals Ethics

The use of the animals was approved by the Institution of Animal Care Committee of the Chinese Academy of Agriculture Sciences (CAAS) (Beijing, China; approval no N40.23, E116.10). Experimental procedures involving feeding and slaughter were approved by the Animal Ethics Committee of the Institute of Feed Research of the CAAS (protocol number: IFR-CAAS-20230611).

The experiment was conducted from June to October 2023, at the experimental station of the Institute of Feed Research of CAAS, Beijing, China.

### 2.2. Animals, Experimental Design and Diets

Twenty-four male Hu lambs with an average age of 2 months old and a body weight of 19.5 ± 1.0 kg were selected at the beginning of the experiment. The lambs were randomly allocated to the following two groups: (1) one group fed with peanut vine (PVH, *n* = 12); (2) and the other group fed with extruded rapeseed straw (ERS, *n* = 12) as the experimental diet. The extrusion of rapeseed straw was performed as follows: each ton of straw was hydrated with 30% of water, the extrusion condition was a temperature range of 150 to 200 °C and a pressure of 15 MPa. Using the dedicated extruder (Model 9P-150, Kunzhentianxi, Inner Mongolia, China), the hourly output of processed rapeseed straw was about 800 kg.

The Hu lambs in each group were fed individually with a basal mixed ration diet, which was formulated to achieve an ADG of 200 g/d for the male lambs according to the national feeding standard. During 0–60 days in the experiment (early stage), the dietary concentrate to roughage ratio was 70:30, while during 60–90 days in the experiment (late stage), the dietary concentrate to roughage ratio switched to 75:25. The ingredients and nutrient contents of the experimental diets as a total mixed ratio (TMR) are shown in Table 1. Each lamb was kept in a separate pen (2.5 m × 1.0 m) and served as a replicate. All lambs were fed twice daily (8:00 am and 6:00 pm) and had ad libitum access to diet (adjusted to less than 10% of refusals daily) and clean water.

### 2.3. Experimental Procedure

The experiment was conducted over a 120-day period, with a 30-day adaptation period. Daily feed intake and average daily feed intake (ADFI) were monitored and calculated by recording the feed offered and refused for each lamb. Lambs were weighed at 30, 60, and 90 days of the experiment to calculate the ADG and feed conversion ratio (FCR).

ADFI (g/d) = Total Feed Intake/Days

ADG (g/d) = (Final Body Weight − Initial Body Weight)/Days

FCR = (ADFI/ADG)

### 2.4. Sample Collection

Rumen fluid. At the end of the experimental period (day 90), as a lamb became ill and was excluded to avoid biased or unreliable data, ten lambs were randomly selected from each group. The selected lambs were fasted for 12 h with free access to water before slaughter. After exsanguination, the rumen was removed and opened. Two 10 mL tubes of rumen fluid were collected by squeezing ruminal digesta from the dorsal sac through four layers of cheesecloth. The pH of one tube was measured immediately.

Serum. Prior to slaughter (7:00 a.m.), blood samples (10 mL) were obtained from the jugular vein of each lamb using centrifuge tubes containing EDTA as an anticoagulant and stored at 4 °C.

Liver. Liver tissue samples (1 cm × 1 cm × 1.5 cm) were collected after slaughter, with three pieces placed in each 2 mL cryovial and stored at –80 °C for the determination of liver metabolites.

Muscle. In addition, approximately 200 g of longissimus thoracic (LT) meat was sampled between the 11th and 12th ribs on the right side of each carcass. The meat samples were stored at −20 °C for further analysis. All samples from each lamb were collected within 10 min post-slaughter.

### 2.5. GC–MS Based Metabolomics Procedure

Rumen fluid and serum samples were prepared as follows. Firstly, 350 μL methanol and 50 mL L-2-chlorophenylalanine were added to 100 μL biofluid samples. The mixture was vortexed, then centrifuged at 4 °C and 12,000 rpm for 10 min. A 0.35 mL aliquot of the supernatant was transferred to a 2 mL silylated vial. After the extracts were dried in a vacuum concentrator, 80 μL o-methyl hydroxylamine hydrochloride was added and gently mixed. The solution was incubated at 37 °C for 2 h. Subsequently, 100 μL bistrifluoroacetamide (containing 1% TCMS, *v*/*v*) was added to each sample, followed by incubation at 70 °C for 1 h. The samples were then analyzed by GC-TOF/MS.

Liver and longissimus lumborum samples were pretreated and determined by a gas chromatograph–mass spectrograph (GC–MS) following a standard method from our previous study [12]. In general, 10 g of sample was added with 2.5 μL internal standard solution (o-dichloro benzene, 0.5 μg/mL in methanol) in the extraction headspace flask with 2 g of sodium chloride heated at 120 °C for 30 min after sealing. Then, the system was cooled to room temperature and taken in a water bath at 60 °C. A solid-phase microextraction (SPME) injector with a 65 pm polydimethylsiloxane vinylbenzene-coated extraction head (Supelco, Bellefonte, PA, USA) was used to fully absorb the volatile flavor substances for 30 min after inserting them into the headspace flask. Then, the extracted SPME needle tube was inserted into the injection port of a gas chromatograph–mass spectrograph (GC–MS, 890B-5977B, Agilent, Palo Alto, CA, USA), thermally desorbed for 3 min, and then stored for later GC-MS analysis.

### 2.6. Statistical Analysis

The ADG, ADFI, and rumen fermentation parameters were analyzed using SAS software (version 9.4, SAS Institute Inc., Cary, NC, USA). A mixed procedure was used as follows:

Y_ijk_ = μ + G_i_ + P_j_(D_j_) + GP_ij_(GD_ij_) + C_k_ +e_ijk_

where Y_jik_ is the dependent variable, μ is the overall mean, G_i_ is the effect of the ith group, P_j_(D_j_) is the effect of the jth period (day), GP_ij_(GD_ij_) is the interaction between group and period (group and day), C_k_ is the random effect of kth lamb, and e_ijk_ is the residual error. Spearman’s rank correlation analysis was performed for all correlation assessments in this study, with the statistical significance threshold set at |r| > 0.50 and *p* < 0.05.

Matrices comprising the relative concentrations of conserved 266 metabolites detected in four biological fluids and tissues were uploaded to the web-based analytical tool MetaboAnalyst (http://www.metaboanalyst.ca/) for integrated pathway analysis. Analysis was performed against the Bos taurus pathway library, combining global test enrichment analysis and relative-betweenness centrality topology analysis. Enriched pathways were visualized in the metabolome view according to enrichment *p*-values and topology-derived pathway impact scores.

Multivariate statistical analysis was performed using SIMCA-P+ 14.1 (Umetrics, Umea, Sweden), including principal component analysis (PCA), partial least-squares discriminant analysis (PLS-DA), and orthogonal partial least-squares discriminant analysis (OPLS-DA). PCA was used to visualize the dataset structure and inter-sample similarities and differences. The PLS-DA model was validated using 7-fold permutation testing. OPLS-DA was applied to maximize covariance between analytical data and response variables. Milk and serum datasets were normalized by mean-centered scaling (Ctr), whereas the rumen fluid samples were processed using unit variance scaling (UV).

OPLS-DA was applied to identify metabolites significantly differing between the ERS and PVH groups. Variable importance in the projection (VIP) scores was generated for the predictive component; metabolites with VIP > 1.0 were initially retained as differential candidates. Remaining variables were further assessed by Student’s *t*-test, and variables with *p* > 0.05 were excluded. Fold change (FC) was calculated as the ratio of mean peak areas between the ERS and PVH groups. Differential metabolites were annotated and verified using KEGG, BMDB, PubChem Compound, ChEBI, NIKKAJI, and CAS. Metabolites were subsequently mapped to KEGG pathways, and the most significantly altered pathways were identified and constructed based on functional enrichment analysis.

## 3. Results

### 3.1. Intake, Growth Performance and Serum Parameters

**Figure 1:**
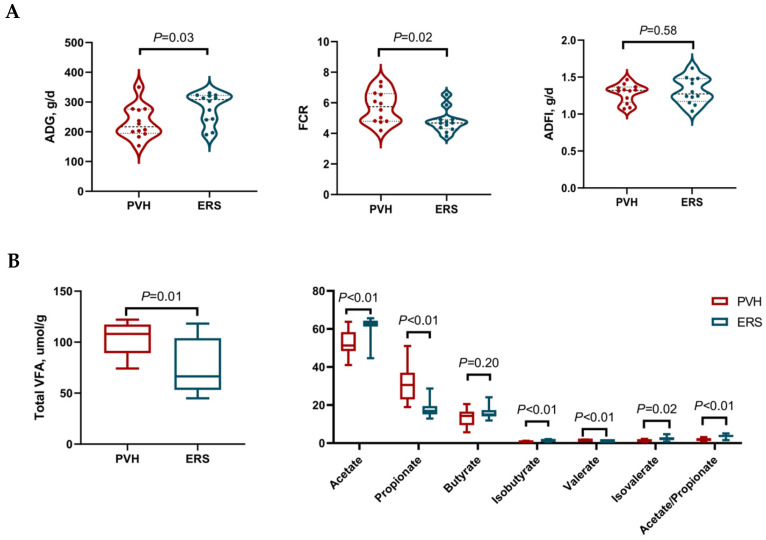
Comparison of phenotypes between PVH and ERS Hu lambs during 60–90 d of the experiment. (**A**) Differences in average daily gain (ADG), average daily feed intake (ADFI), and feed conversion ratio (FCR) between PVH and ERS Hu lambs; (**B**) differences in rumen fermentation between PVH and ERS Hu lambs; PVH, peanut vine hay group; ERS, extruded rapeseed straw group.

The growth performance of Hu lambs has been previously reported [2]. In short, there is no difference in BW, DMI, ADG and FCR overall during 0–90 days between the ERS and PVH group, while no significant treatment × day interaction was observed for BW and DMI. However, there is an interaction between dietary treatment and time, as ADG was significantly greater in Hu lambs in the ERS group compared with the PVH group during 60–90 days in the experiment (*p* = 0.03); also, the ERS group showed a better FCR performance than the PVH group (Figure 1A). However, the ADFI did not differ between the two groups (*p* > 0.05; Figure 1A).

### 3.2. Ruminal Fermentation Parameters Concerning VFAs

Remarkably, lower concentrations of total VFAs, lower proportions of propionate and valerate and higher proportions of acetate, isobutyric and isovaleric acid were detected in the rumen of lambs fed with ERS than those fed PVH (*p* < 0.05); also, the values of acetate/propionate in the rumen of lambs fed with ERS were higher than those fed with PVH (*p* < 0.05) (Figure 1B). No other significant differences in fermentation parameters were observed between the two groups (*p* > 0.05) (Figure 1B).

### 3.3. Metabolic Pathway of Common Metabolites

**Figure 2:**
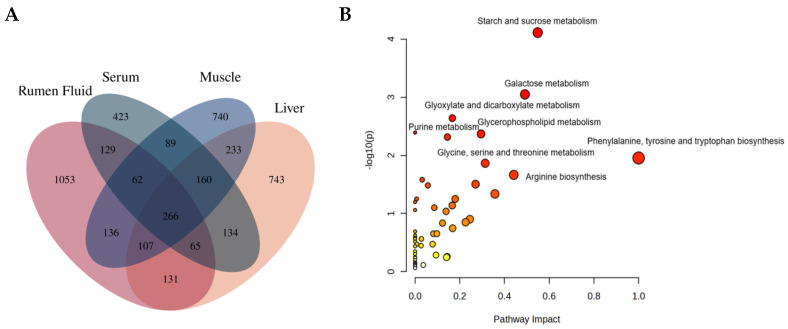
Unique and mutual metabolites identified in the rumen fluid, serum, liver and muscle of Hu lambs (**A**). Metabolome view map of the common metabolites identified in four biofluids from dairy cows fed ERS and PVH diets (**B**). The x-axis represents the pathway impact, and y-axis represents the pathway enrichment. Larger sizes and darker colors represent higher pathway enrichment and higher pathway impact values, respectively. PVH, peanut vine hay group; ERS, extruded rapeseed straw group.

As shown in Figure 2, the metabolome view map revealed that the enriched pathways (*p* < 0.05) for 266 metabolites identified in all four biological matrices were starch and sucrose metabolism, galactose metabolism, glyoxylate and dicarboxylate metabolism, glycerophospholipid metabolism, phenylalanine, tyrosine and tryptophan biosynthesis, phenylalanine metabolism, Gly, Ser, and Thr metabolism, arginine biosynthesis, Butanoate metabolism, Histidine metabolism and purine metabolism. Eight of them had a pathway impact value higher than 0.1, which is the cutoff value for relevance. The impact values of starch and sucrose metabolism, galactose metabolism, glyoxylate and dicarboxylate metabolism, glycerophospholipid metabolism, phenylalanine, tyrosine and tryptophan biosynthesis, glycine, serine and threonine metabolism, arginine biosynthesis, and purine metabolism were 0.54, 0.49, 0.17, 0.30, 0.99, 0.31, 0.44, and 0.27 respectively. Based on both the *p*-value and impact value, these eight pathways were characterized as the significantly relevant pathways.

### 3.4. Identification of Significant Differential Metabolites

**Figure 3:**
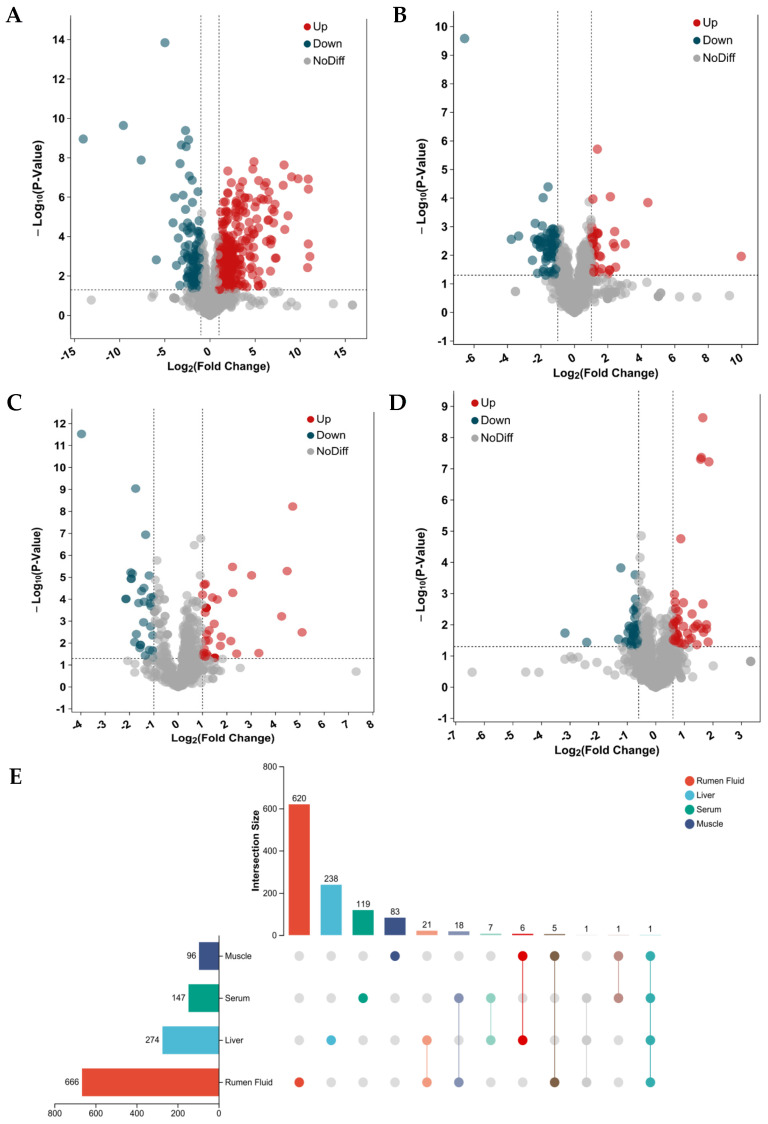
Volcano of differential metabolites of rumen fluid (**A**), liver (**B**), serum (**C**) and muscle (**D**) for Hu lambs fed PVH and ERS. Venn plot of shared differential metabolites in rumen fluid, liver, and serum for Hu lambs fed PVH and ERS (**E**). PVH, peanut vine hay group; ERS, extruded rapeseed straw group.

In total, 666, 274, 147 and 96 metabolites were quantified in the rumen fluid, liver, serum and muscle, of which 620, 238, 119 and 83 metabolites were unique to each corresponding biological matrix, respectively (Figure 3). The metabolites such as alpha-Ketoisovaleric acid, 5-Hydroxyhexanoic acid, 3-Methylglutaric acid, and 617 others that were produced by rumen fermentation were only detected in the rumen fluid, whereas metabolites involved in liver-specific processes such as detoxification, lipid metabolism, and amino acid metabolism, including 1-Palmitoyl-2-azelaoylphosphatidylcholine, 17beta-Estradiol 3-beta-D-glucuronide, gamma-Glutamyltyramine, Palmitoylcarnitine (Car (16:0)), and 270 other differential metabolites, were only identified in the liver. Metabolites such as Histidine, pyruvate, Glycocholic acid, Hydroxyphenyllactic acid, and 143 other metabolites were only detected in the serum, and Eicosapentaenoic acid, PC (31:1), PC (22:2), PC (38:3), and 92 other compounds were only identified in the muscle. In addition, Hypaphorine was identified in all four biological matrices, and N-Methyltrimethylacetamide was identified in the rumen fluid, liver and serum. Meanwhile, 21 were common in the rumen fluid and liver, 18 were common in the rumen fluid and serum; six in the liver and muscle, and five in the rumen fluid and muscle. The numbers of metabolites that were identified are illustrated in Figure 3.

### 3.5. Significantly Different Metabolites and Key Different Metabolic Pathways Between the Two Diets

In total, nine, twelve, seven, and three significantly different metabolites (VIP > 1 and *p* < 0.05) related to eight significant pathways were identified in the rumen fluid, liver, serum, and muscle, respectively, between the ERS and PVH diets (Table 2). The fold change (FC) value was used to indicate the specific, variable quantity in the ERS diet compared with the PVH diet. As shown in Table 2, in the rumen fluid, metabolites belonging to galactose metabolism, starch and sucrose metabolism and carbohydrate digestion and absorption including sucrose (FC = 4.96), mannose (FC = 2.04), glucose (FC = 2.04), tagatose (FC = 2.36), lactose (FC = 3.35), melibiose (FC = 2.50), maltose (FC = 4.96), cellobiose (FC = 2.90), and isomaltose (FC = 2.90) were all lower in the ERS group compared with the PVH group; in the liver, three arginine biosynthesis metabolites, three glycerophospholipid metabolism and metabolites and six purine metabolism metabolites like adenosine, deoxyguanosine, PAP, xanthosin, uric acid. For significantly different metabolites in the serum including pyruvate, 3-Phenylpropanoic acid, phenylacetylglutamine, and so forth, five metabolites were found at higher concentrations in the ERS group than in the PVH group. In the muscle, all three metabolites belonging to purine metabolism were found at higher concentrations in the ERS group than in the PVH group.

### 3.6. Correlation Analysis of Significant Different Metabolites and Growth and Rumen Fermentation Parameters

Upon further analysis between significantly different metabolites in the four biological matrices and rumen fermentation and growth parameters, all the metabolites in the rumen fluid, including mucrose, mannose, glucose, tagatose, lactose, melibiose, maltose, cellobiose, and isomaltose, exhibited positive correlations with the total VFAs, propionate and valerate concentrations (R > 0.50, *p* < 0.05), and negative correlations with the acetate, isobutyrate and isovalerate concentrations. In the liver, metabolites related to arginine biosynthesis including N-acetylglutamic acid and glutamate are positively related with propionate concentration and negatively related to the acetate concentration. 3-Amino-3-(4-hydroxyphenyl) propanoic acid in the serum is positively related to the acetate concentration but negatively related to the propionate concentration. The three significantly different metabolites in the muscle including ADP, PAP and dGDP are positively related to the acetate and isobutyrate concentrations, negatively related to the total VFAs and propionate concentrations.

**Figure 4:**
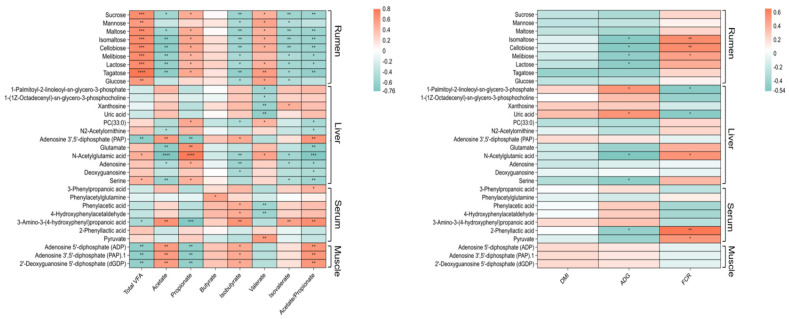
Relationships of differential metabolites of rumen fluid, liver, serum and muscle for Hu lambs fed PVH and ERS with growth (ADG, DMI, ADFI) and rumen fermentation (VFAs) parameters. * means Spearman’s |r| > 0.50 and *p* < 0.05, **, ***, **** means Spearman’s |r| > 0.50 and *p* < 0.01. PVH, peanut vine hay group; ERS, extruded rapeseed straw group.

**Figure 5:**
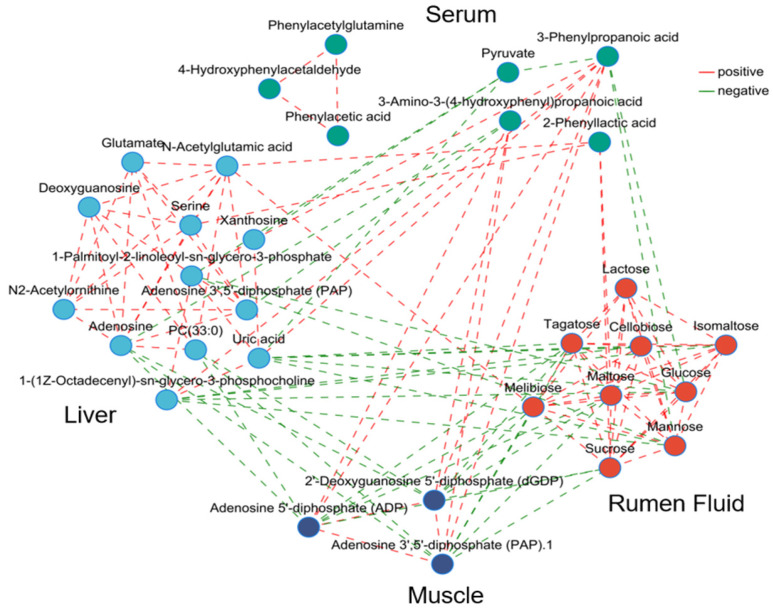
Network analysis to reveal metabolites interactions in the four fluids and tissues. The network analysis showed the degree of correlation between the significant different metabolites in rumen fluid, serum, liver and muscle (Spearman’s |r|> 0.50 and adjusted *p* < 0.05). Lines between two nodes represent the correlation, with a red line indicating a positive correlation and a green line indicating a negative correlation.

About the correlations between different metabolites and growth parameters, lactose, melibiose, cellobiose and isomaltose displayed positive correlations with FCR and negative correlations with ADG. In the liver, 1-Palmitoyl-2-linoleoyl-sn-glycero-3-phosphate and uric acid were positively related to ADG and negatively related to FCR. In contrast, N-acetylglutamic acid was negatively related to ADG and positively related to FCR. Only 2-Phenyllactic and pyruvate in the serum showed positive correlations with FCR, and 2-Phenyllactic was positively related to ADG. There was no significant correlation between metabolites in the muscle and growth parameters (Figure 4). Correlations between significant metabolites in the four fluids and tissues suggest a rich metabolic association in the network (Figure 5).

## 4. Discussion

In this study, we characterized the changes in growth performance and rumen fermentation of Hu lambs in response to replacing PVH with ERS, and metabolomics analysis was used to compare the metabolites and to detect their changes in the rumen fluid, serum, liver, and muscle tissues from Hu lambs under two types of forage, aiming to explore the potential utilization of the ERS and discover potential biomarkers for ADG in Hu lambs under ERS feeding.

### 4.1. Growth Performance and Rumen Fermentation

The significantly increased ADG with the improved FCR in the ERS group indicates a positive effect on the growth performance of the ERS diet in Hu lambs. Normally, feed intake is the dominant factor that could influence ADG [13]; we did not observe any differences in ADFI between the two groups in our study, suggesting other possible factors are responsible for the difference in nutrient utilization and increased ADG of the Hu lambs in our study.

About the rumen fermentation, the total VFAs and proportion of propionate were reduced, while the proportion of acetate and the acetate-to-propionate (A:P) ratio were significantly increased in the ERS group compared with the PVH group. The ratio of A:P among VFAs in the rumen directly affects the efficiency of energy conversion [14,15]. Energy utilization efficiency can be enhanced by optimizing the A:P ratio [16]. Therefore, a greater ADG in our study is a possible result from the optimization of fermentation product proportions and more balanced fermentation [17], which is specifically reflected in the directional changes in key VFAs (higher proportion of acetate and lower proportion of propionate in particular) and their subsequent regulatory effects on systemic metabolism.

### 4.2. Metabolites and Metabolic Pathways

For lambs fed with the ERS diet, galactose metabolism, starch and sucrose metabolism and carbohydrate digestion and absorption in the rumen was negatively altered, indicated by the downregulated-related metabolites including sucrose, mannose, glucose, tagatose, lactose, melibiose, maltose, and isomaltose. The metabolites are all non-structural carbohydrates and their degradation products belong to rapidly fermentable types. Metabolic changes indicate that the core fermentation substrate of the ERS group may be the slowly fermented structural carbohydrates. Fermentation of structural carbohydrates depends on specific microorganisms (e.g., fiber-degrading bacteria), and their metabolic products are dominated by acetate [16,18]. As fermentable carbohydrates (the substrate for propionate production) decreased, the concentrations of total VFAs and propionate decreased synchronously [19]. Propionate is converted to glucose in the liver, which is mainly used for maintenance metabolism [20]. In contrast, the proportion of acetate increased. Acetate is a high-quality energy source for body fat deposition and muscle growth [21], with a higher energy utilization efficiency, thereby increasing the net energy available for growth.

The hepatic metabolic profile in our study exhibited adaptive adjustments to match the ruminal fermentation shifts. Firstly, the downregulation of arginine biosynthesis, indicated by decreased N-acetylglutamic acid, N2-acetylornithine, and glutamate, reduced energy consumption for maintenance metabolism [22], redirecting nutrients to growth-related anabolism. Secondly, the remodeling of glycerophospholipid metabolism, including increased PC (33:0) and decreased 1-(1Z-octadecenyl)-sn-glycero-3-phosphocholine, enhanced the fluidity of hepatic cell membranes [23], promoting the transport efficiency of acetate and amino acids. Thirdly, the bidirectional regulation of purine metabolism balanced nucleic acid synthesis for growth-related tissues in the liver and muscle while reducing invalid consumption for microbial proliferation [24]. Collectively, these hepatic metabolic adjustments optimized nutrient allocation and energy utilization.

Serum metabolic changes in our study further confirmed the improved nutrient supply and metabolic homeostasis in the ERS group. Chen et al. (2006) found a significant increase in the A:P ratio occurred with the infusion of high-concentration pyruvate [25], which is similar to our study. The increased 3-phenylpropionic acid and phenylacetylglutamine indicated the enhanced conversion efficiency of ruminal acetate to pyruvate in the liver [23], providing sufficient energy substrates for muscle tissue; and optimized rumen–intestinal microbial homeostasis reflected by elevated aromatic metabolites, which strengthened intestinal barrier function and improved nutrient absorption integrity.

In the current study, muscle metabolism presented direct molecular evidence for ADG improvement, with a key focus on purine metabolism. Muscle protein synthesis is an energy-intensive process that requires ATP to drive key steps such as amino acid activation and peptide chain elongation [26]. Enhanced purine metabolism promotes increased ATP synthesis, which directly provides sufficient energy for muscle protein synthesis, thereby facilitating muscle deposition and ultimately contributing to the improved ADG of finishing Hu lambs. Therefore, the increased concentrations of ADP, dGDP, and PAP in the ERS group directly supported muscle growth. In addition, ADP enhanced energy reserves for muscle fiber synthesis by serving as a direct precursor for ATP [27]; dGDP promoted muscle cell proliferation and fiber repair by providing essential raw materials for DNA synthesis [28]. Notably, these muscle purine metabolites were negatively correlated with the total VFAs and propionate concentrations but positively correlated with the acetate concentration, forming a downstream synergistic effect of ruminal fermentation optimization. Specifically, the increased acetate proportion provided sufficient acetyl-CoA for muscle energy metabolism and carbon skeletons for muscle synthesis [29], while reduced propionate minimized glucose consumption for maintenance, indirectly facilitating purine synthesis in the muscle. Purine metabolism has also been conformed in improving umami taste [30].

### 4.3. Integrated Pathways

An increased proportion of acetate contributes substantially to the improved growth performance. Acetate is typically considered a low-energy VFA relative to propionate; however, its elevated level in the ERS group exerted a pivotal positive effect on energy utilization efficiency. As the primary end product of ruminal microbial fermentation, acetate can be efficiently transported to skeletal muscle through the systemic blood circulation [31,32]. Upon reaching the muscle tissue, acetate undergoes β-oxidation in mitochondria to generate ATP, which directly provides the energy required for muscle protein synthesis. This regulatory pathway is strongly corroborated by the correlation analysis results: a significant positive correlation was observed between the muscle ADP content and ruminal acetate proportion. The increased ADP level indicates an accelerated ATP synthesis process in muscle cells, which confirms that acetate serves as an effective energy substrate to fulfill the energy demand of muscle growth.

A decreased proportion of propionate, coupled with the downregulated ruminal carbohydrate metabolites, further clarifies the optimized energy utilization pattern induced by the dietary intervention. Traditionally, propionate is recognized as the major precursor for hepatic gluconeogenesis in ruminants. Despite decreased propionate production, glucose homeostasis was maintained in lambs fed with extruded rapeseed straw, likely via enhanced gluconeogenesis from amino acids as an alternative substrate. This metabolic shift reduced reliance on propionate-driven glucose synthesis while improving overall energy efficiency, thereby supporting a normal glucose balance and greater average daily gain.

However, excessive propionate production would impose a heavy metabolic burden on the liver. Specifically, surplus propionate would be rapidly converted into glucose, leading to transient hyperglycemia. The excess glucose that cannot be immediately utilized for energy supply or glycogen storage would be converted into triglycerides and deposited in adipose tissue, thereby competing with muscle tissue for energy substrates and inhibiting muscle protein deposition. In this study, the reduced propionate proportion alleviated the metabolic load of hepatic gluconeogenesis, effectively avoiding the energy waste caused by excessive fat deposition induced by short-term hyperglycemia. This stable energy supply with a low waste mode not only ensures the continuous energy input for muscle growth but also reduces the energy consumption in non-productive metabolic processes, which may jointly contribute to the improvement in ADG.

## 5. Conclusions

**Figure 6:**
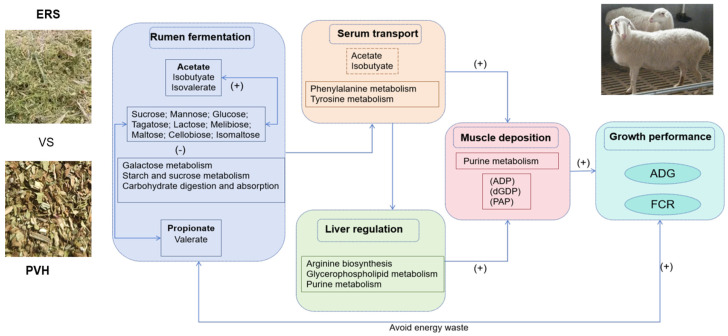
Hypothesized scheme pathways and potential mechanisms related to the changes in the ADG under the PVH and ERS feeding. (+) and (−) mean the changes in metabolites and pathways of the biological matrices under PVH and ERS feeding. PVH, peanut vine hay group; ERS, extruded rapeseed straw group.

In our study, feeding ERS to Hu lambs instead of PVH constructs a synergistic regulatory network (Figure 6) across the rumen, liver, serum, and muscle. The core driver is the shift in ruminal fermentation from non-structural carbohydrate-dominated rapid fermentation to structural carbohydrate-dominated steady fermentation, which is further amplified by metabolic adaptations in fluid and tissue. Compared with PVH feeding, ERS feeding reduced the invalid nutrient consumption indicated by decreased ruminal microbial sequestration and hepatic maintenance metabolism, enhanced nutrient transport and utilization through hepatic glycerophospholipid remodeling and serum substrate supply, and strengthened muscle anabolism. This integrated metabolic optimization ultimately leads to improved ADG in the ERS feeding group. These findings provide a theoretical basis for the rational utilization of extruded rapeseed straw as a ruminant feed and deepen the understanding of the link between dietary roughage sources and overall metabolic regulation.

## Figures and Tables

**Table 1:** Composition and nutrient levels of experimental diets (air dry basis, %).

Dietary Ingredients (%)	Early Stage		Late Stage	
Groups	PVH	ERS	PVH	ERS
Peanut vine	30.0	-	25.0	-
Extruded rape straw	-	30.0	-	25.0
Corn	41.0	41.0	46.0	46.0
Soybean meal	15.0	15.0	15.0	15.0
Wheat bran	10.0	10.0	10.0	10.0
Premix ^1^	4.0	4.0	4.0	4.0
Total	100.0	100.0	100.0	100.0
Chemical composition				
ME (MJ/kg) ^2^	12.3	12.3	12.6	12.6
Dry matter (%)	86.0	86.0	86.0	86.0
Crude protein (%)	12.9	12.5	12.9	12.6
EE (%)	2.6	2.6	2.6	2.7
NDF (%)	29.9	30.7	29.9	28.1
ADF (%)	19.6	20.1	19.6	17.9
Ca (%)	1.1	1.1	1.1	1.1
P (%)	0.5	0.5	0.5	0.5

ME = metabolizable energy; EE = ether extract; NDF = neutral detergent fiber; ADF = acid detergent fiber. PVH = peanut vine hay; ERS = extruded rapeseed straw. ^1^ The premix provided the following per kg of diets: VA 15 000 IU, VD 2200IU, VE 50 IU, Fe 55 mg, Cu 12.5 mg, Mn 47 mg, Zn 24 mg, Se 0.5 mg, Co 0.1 mg. ^2^ ME (MJ/kg) was calculated as ME intake (MJ/d)/DMI (kg/d) during the digestibility trial.

**Table 2:** Metabolic pathways identified from the significantly different metabolites (SDMs), the rumen fluid, liver, serum and muscle of the Hu lambs, between PVH and ERS diets.

	Metabolic Pathways	SDMs
Rumen Fluid	Gal, StSuc, Carb ^a^	(4.96) sucrose; (2.04) mannose; (2.04) glucose; (2.36) tagatose; (3.35) lactose; (2.50) melibiose; (4.96) maltose; (2.90) cellobiose; (2.90) isomaltose;
Liver	Arg, GPLipid, Pur ^b^	(1.67) N-acetylglutamic acid; (1.53) N2-Acetylornithine; (1.66) glutamate; (0.54) 1-(1Z-Octadecenyl)-sn-glycero-3-phosphocholine; (1.51) PC (33:0); (0.35) 1-Palmitoyl-2-linoleoyl-sn-glycero-3-phosphate; (2.12) serine; (1.74) adenosine; (1.91) deoxyguanosine; (0.55) xanthosin; (0.60) uric acid; (1.56) Adenosine 3,5-diphosphate (PAP);
Serum	Phe, Tyr ^c^	(0.32) pyruvate; (0.51) 3-Phenylpropanoic acid; (0.58) phenylacetylglutamine; (0.59) Phenylacetic acid; (2.84) 2-Phenyllactic acid; (0.59) 3-Amino-3-(4-hydroxyphenyl) propanoic acid; (0.59) 4-Hydroxyphenylacetaldehyde;
Muscle	Pur ^d^	(0.60) Adenosine 5-diphosphate (ADP); (0.58) 2-Deoxyguanosine 5-diphosphate (dGDP); (0.60) Adenosine 3,5-diphosphate (PAP).

^a^ Gal, StSuc, Carb = galactose metabolism, starch and sucrose metabolism and carbohydrate digestion and absorption; ^b^ Arg, GPLipid, Purb = arginine biosynthesis, glycerophospholipid metabolism and purine metabolism; ^c^ Phe, Tyr = phenylalanine metabolism and tyrosine metabolism; ^d^ Pur = purine metabolism; PVH, peanut vine hay group; ERS, extruded rapeseed straw group.

## Data Availability

The raw datasets used and analyzed during the current study are available from the corresponding author upon reasonable request.

## Abbreviations

The following abbreviations are used in this manuscript:

ERS	Extruded rapeseed straw
PVH	Peanut vine hay
ME	Metabolizable energy
CP	Crude protein
EE	Ether extract
NDF	Neutral detergent fiber
ADF	Acid detergent fiber
ADFI	Average daily feed intake
ADG	Average daily gain
DMI	Dry matter intake
FCR	Feed conversion ratio
TMR	Total mixed ratio
VFAs	Volatile fatty acids
LT	Longissimus thoracic
A:P	Acetate-to-propionate ratio
PAP	Adenosine 3,5-diphosphate
ADP	Adenosine 5-diphosphate
dGDP	Deoxyguanosine 5-diphosphate
